# Total hip arthroplasty with femoral osteotomy and modular prosthesis for proximal femoral deformity

**DOI:** 10.1186/s13018-019-1336-1

**Published:** 2019-08-29

**Authors:** Xiaowen Deng, Jun Liu, Tao Qu, Xusheng Li, Ping Zhen, Qiuming Gao, Yun Xue, Peng Liu, Guoding Cao, Xiaole He

**Affiliations:** 1 Department of Joint Surgery, Institute of Orthopedics, 940th Hospital of PLA Joint Logistics Support Force, Lanzhou, 730050 China; 2 Department of Geriatrics Center, Xijing Hospital of Air Force Military Medical University, Xi’an, 710032 China

**Keywords:** Proximal femoral deformity, Femoral osteotomy, Total hip arthroplasty, Modular S-ROM prosthesis, Femoral reconstruction

## Abstract

**Background:**

Severe anatomical abnormalities exist in proximal femoral deformities (PFDs). Total hip arthroplasty (THA) is associated with drawbacks such as high surgical complexity, long operation time, requirement for high surgical skills, high incidences of postoperative complications, and poor efficacy.

**Objective:**

This study aimed to investigate the short-term efficacy of THA with femoral osteotomy and modular prosthesis implantation for femoral fixation and reconstruction in patients with PFD.

**Methods:**

A total of 15 patients (15 hips) with rotational PFD treated with THA with femoral osteotomy and modular prosthesis between August 2012 and September 2014 were included. There were 10 male (10 hips) and 5 female (5 hips) patients. Preoperative limb shortening, intraoperative osteotomy length, and postoperative limb length were recorded. The Harris hip score was adopted for assessing the clinical results. Postoperative radiography was performed to observe the prosthesis position, as well as the presence or absence of abnormalities such as osteolysis, loosening, and subsidence of the prosthesis.

**Results:**

All 15 patients were followed up postoperatively, with a mean follow-up duration of 62.5 (range 20–85) months. The postoperative limb-length discrepancy (1.0 ± 0.5 cm) was significantly less than the preoperative discrepancy (3.2 ± 1.2 cm) (*t* = − 2.501, *P* = 0.002). The Harris hip score significantly improved from a mean of 47.2 ± 9.9 points preoperatively to 89.7 ± 3.9 points during the last follow-up visit (*t* = 21.31, *P* = 0.001). Immediate postoperative radiographs showed restoration of limb alignment after femoral osteotomy, excellent initial press-fit fixation of the S-ROM prosthesis, and good canal filling. According to Engh’s criteria, all 15 hips were graded as ingrown bones. No infection, prosthesis loosening, periprosthetic fracture, or other complications occurred.

**Conclusion:**

In patients with femoral deformities treated with THA, precise osteotomy, good coaptation of the osteotomy surfaces, and correct choice of modular S-ROM prostheses for femoral reconstruction and fixation remain the key factors for surgical success.

## Background

Proximal femoral deformity (PFD) in adults can be induced by several causes, including congenital hip disease, proximal femoral infection, trauma, bone disease, and history of proximal femoral osteotomy [1–5]. Total hip arthroplasty (THA) remains challenging in patients with PFD complicated with femoral head necrosis or end-stage hip disease [1, 5]. In patients with rotational PFD, femoral osteotomy is required for the correction of limb alignment. Therefore, precise osteotomy, good coaptation of the osteotomy surfaces, and correct choice of the modular S-ROM prostheses for femoral reconstruction and fixation are the key factors for surgical success [1, 6–8].

In this study, 15 patients with rotational PFD were treated with THA combined with femoral osteotomy and modular prosthesis implantation and fixation between August 2012 and September 2014. We herein aimed to investigate (1) the methods of femoral osteotomy correction and fixation for PFD, (2) the key surgical techniques and precautions when using the S-ROM prosthesis, and (3) the short-term clinical efficacy of THA with femoral osteotomy and modular prosthesis implantation for PFD.

## Methods

### General data

In this study, a total of 10 male patients (10 hips) and 5 female patients (5 hips), with a mean age of 43.5 (range 38–52) years were enrolled. There were 7 cases of left hips and 8 cases of right hips. All 15 cases had end-stage hip disease complicated with subtrochanteric PFD, including 6 cases with developmental dysplasia of the hip (DDH), 5 cases with a post-femoral osteotomy (for congenital hip dislocation) status, 3 cases with malunion after a proximal femoral fracture, and 1 case with old septic hip arthritis. The chief complaints of the patients included hip pain aggravated with activities or weight-bearing, as well as claudication (9 cases of severe claudication and 6 cases of moderate claudication). Limb discrepancy was observed in all 15 patients, with a mean shortening of 3.2 (range 2.0–4.5) cm in the affected leg. A positive Trendelenburg sign was observed in all patients, with 5 patients having grade 5, 9 patients having grade 4, and 1 patient having grade 3 gluteus medius strength (Table 1). The preoperative Harris hip score was 41.00 ± 6.38 points. Tests for erythrocyte sedimentation rate and C-reactive protein, as well as a radionuclide scan, were performed to rule out possible active infections in 1 patient with old septic hip arthritis.

**Table 1:** Baseline data of 15 patients

Disease type	Patient	Sex	Age	Geometry type	Degree of claudication	Degree of shortening	Glutaeus medius strength	Length of femoral osteotomy	Follow-up duration
DDH (Crowe type IV)	No.1	Male	45	Rotational	Severe claudication	Severe shortening	Grade 4	3.5	49
	No.2	Male	39	Rotational with canal occlusion	Severe claudication	Severe shortening	Grade 5	3.3	58
	No.3	Male	49	Rotational	Moderate claudication	Moderate shortening	Grade 4	2	45
	No.4	Female	46	Rotational	Moderate claudication	Moderate shortening	Grade 4	2.1	85
DDH (Crowe type III)	No.5	Female	48	Rotational with horizontal displacement	Moderate claudication	Moderate shortening	Grade 4	2.2	52
	No.6	Male	48	Rotational	Moderate claudication	Moderate shortening	Grade 5	2.3	20
Malunion following proximal femoral fracture	No.7	Male	43	Rotational	Severe claudication	Severe shortening	Grade 4	4.2	57
	No.8	Female	48	Rotational with horizontal displacement	Severe claudication	Severe shortening	Grade 4	3.5	65
	No.9	Male	47	Rotational with horizontal displacement	Severe claudication	Severe shortening	Grade 4	3.7	71
Post femoral osteotomy for congenital hip dislocation	No.10	Female	50	Rotational with horizontal displacement	Severe claudication	Severe shortening	Grade 4	3.1	65
	No.11	Male	52	Rotational	Moderate claudication	Moderate shortening	Grade 5	2.5	49
	No.12	Male	44	Rotational	Moderate claudication	Moderate shortening	Grade 5	2.6	72
	No.13	Female	48	Rotational with horizontal displacement	Severe claudication	Severe shortening	Grade 4	3.5	80
	No.14	Male	43	Rotational with canal occlusion	Severe claudication	Severe shortening	Grade 3	3.8	70
Old septic hip arthritis	No.15	Male	41	Rotational	Severe claudication	Severe shortening	Grade 4	3.8	56

Note: *DDH* developmental dysplasia of the hip

### Imaging examinations

Anteroposterior (AP) pelvic radiographs, together with rolled lateral hip and full-length femoral radiographs of the affected side, were taken for each patient to understand the anatomical position and morphology of the femoral deformity. Computerized tomography of the hip and proximal femur was performed to assess the morphology of the acetabulum and the stenosis of the proximal femoral canal. Berry’s classification for PFD was adopted, as follows [5]: (1) based on the cause of deformity: DDH (6 hips, including 4 Crowe [9] type IV DDH and 2 Crowe type III DDH), malunion after proximal femoral fracture (3 hips), old septic hip arthritis (1 hip), and subtrochanteric osteotomy after congenital hip dislocation (5 hips); (2) based on the site of deformity: below the greater trochanter (all 15 cases of rotational PFD); and (3) based on the geometry of deformity: rotational (8 hips), rotational with horizontal displacement (5 hips), and rotational with canal occlusion (2 hips).

### Treatment methods

#### Preoperative preparations

Measurements were made based on the standard radiograph by using an isometric template. (1) Acetabular preparation: Ranawat’s acetabular triangle was used to determine the approximate rotation center of the acetabulum. Thereafter, a suitable-sized acetabular template was selected and placed such that its lower edge was equalized with the radiographic teardrop, and its medial border was close to Kohler’s line when it was abducted 45°, whereas the maximal coverage should reach the premise of maximal preservation of the bony acetabulum. In patients with DDH, the percentage of coverage of the acetabular component was measured and the need for intraoperative structural bone graft was predicted. (2) Femoral preparation: Initially, the rotation center of the femoral head was marked based on the distance of the contralateral femoral head center. Subsequently, the osteotomy line at the femoral neck and the length of the bony segment required for osteotomy at the deformity were marked. This was followed by coaptation of the osteotomy surfaces, and a femoral template with a suitable canal shape and size was used to reconstruct the femoral offset and to calculate the fixation length of the distal prosthesis. The final femoral length after prosthesis implantation was compared with the data of the contralateral femur to determine the reconstructed length of the affected leg.

#### Surgical technique

Under general anesthesia or continuous epidural anesthesia combined with subarachnoid block anesthesia, the patient was placed in a lateral decubitus position (on the unaffected side). A posterolateral approach was then adopted. After the blunt separation of the gluteus maximus, transection of the hip extensors, and excision of the joint capsule, the femoral head was posteriorly dislocated and osteotomy of the femoral neck was performed at the preoperative planned plane. The location of the acetabulum was determined using the acetabular notch and transverse acetabular ligament. The acetabulum was reamed to a suitable size by using a series of reamers (starting from the smallest size and ending with the largest suitable size). A biological acetabular cup with corresponding size and linings was implanted, while the hip was abducted at 40°–45° and anteverted at 15°–20°. A transverse femoral osteotomy was performed at the most severely deformed site of the subtrochanteric region according to the preoperative plan. The length of osteotomy was determined according to the superior shift of the femur on the AP pelvic radiograph and the preoperative template-measured limb-length discrepancy. In this study, the mean length of osteotomy was 2.7 (range 2–4.2) cm. Then, the proximal and the distal ends of the femur were aligned and a 10-mm-diameter intramedullary metal rod was inserted from the transected femoral neck and forwarded to the canal of the distal femur. A C-arm radiography system was used to assess the coaptation of the osteotomy surfaces. If the coaptation was suboptimal, the alignment angle was adjusted to maximally achieve smooth coaptation of the osteotomy surfaces. Both the proximal and distal ends of the osteotomy were annularly bound and fixed by 2 steel wires to avoid femoral split during reaming. The S-ROM prosthesis was then inserted from the proximal femur to the distal femur. After the implantation of the trial head and neck with an appropriate length and anteversion adjustment, the femoral offset was matched with that of the contralateral side, reducing the hip joint. The range of movement and stability of the hip joint were assessed, and the C-arm radiography system was used to evaluate the intramedullary filling of the prosthesis and the location of the acetabular cup. After withdrawing the trial template, the prosthesis sleeve and stem were inserted successively. The transected deformed femoral segments were longitudinally split into the cortical grids, and these grids were fixed circularly with steel wires, enveloping the osteotomy surfaces [10]. The incision was closed after implanting a suitable femoral head. A dwelling drain was provided and routine intraoperative antibiotic prophylaxis was administered in all patients.

**Fig. 1:**
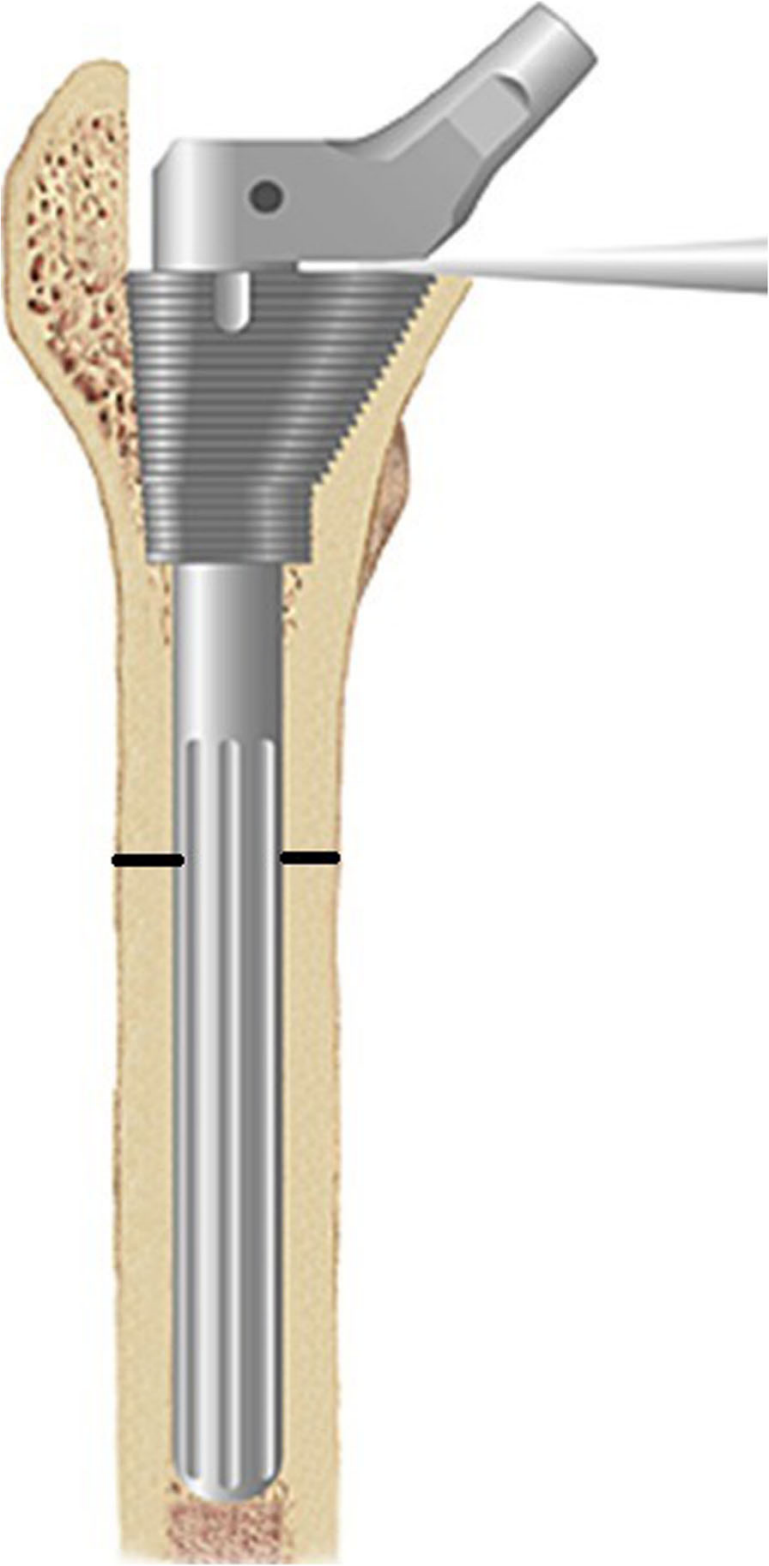
Modular S-ROM prosthesis. The prosthesis consists of a porous-coated sleeve proximally for press-fit fixation and a fluted and slotted stem distally for producing an intramedullary nail fixation effect. The intramedullary filling of the prosthesis distally and proximally can simultaneously achieve stable fixation of the distal and proximal femur

Prosthesis selection: The modular S-ROM prosthesis (Johnson & Johnson, Raynham, MA, USA) was used in all patients. This consists of a proximal porous-coated sleeve for ingrown bones, a stem that is fluted and slotted distally, and a longitudinal ridge structure at the metal surface that provides an anti-rotation stability, which can simultaneously achieve press-fit fixation both proximally and distally. Femoral anteversion could be adjusted by changing the alignment between 2 components of the prosthesis. The 2 parts of the prosthesis are fixed through taper press-fitting, whereas the intramedullary filling of the prosthesis, both distally and proximally, can simultaneously achieve stable fixation of the distal and proximal femur (Fig. 1). Considering the young age of the present study patients, the fourth-generation ceramic-on-ceramic bearing was used in all patients.

#### Postoperative rehabilitation

The affected limb was fixed at 30° abduction, T-shape anti-rotation boots were given, and flexion of the hip and knee was maintained. On day 1 after the operation, the drain was removed and static training of the affected limb was initiated. Thereafter, active and passive flexion and extension exercises of the hip were prescribed. At 3 weeks postoperatively, the patients were encouraged to perform non-weight-bearing walker-aided ambulation. After 6 weeks, partial weight-bearing was allowed. Radiographic examination was performed every month during the follow-up, and full-weight bearing was performed until the union of the fracture.

### Statistical analysis

SPSS 19.0 software (SPSS, USA) was used for statistical analysis. The limb-length discrepancy and Harris hip score before and after surgery were compared using a paired-sample *t* test, with *P* < 0.05 considered to indicate statistical significance.

## Results

**Fig. 2:**
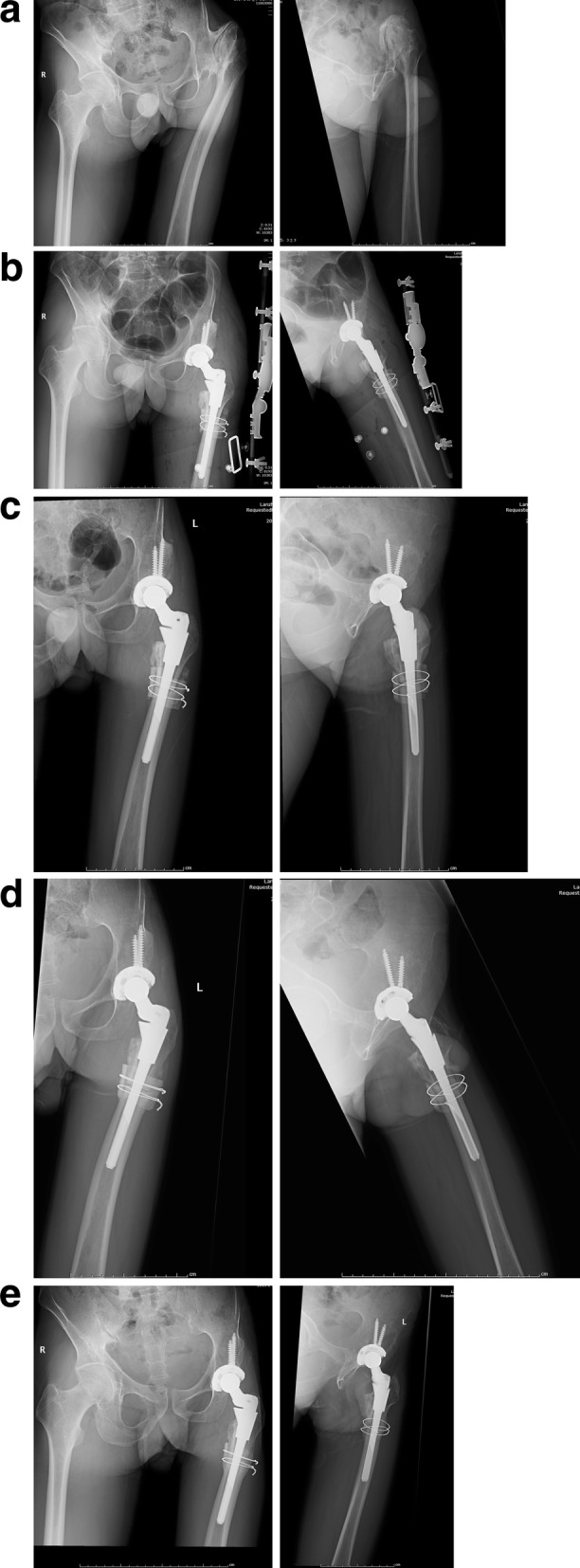
Example case of a 42-year-old male patient. This patient had left Crowe type IV DDH and a Harris hip score of 42 points. **a1**–**a2** Preoperative pelvic radiograph showing significant superior dislocation of the left femoral head forming a joint with a false acetabulum, left DDH, and subtrochanteric PFD. **b1**–**b2** Immediate postoperative AP pelvic and lateral femur radiographs after total hip arthroplasty with osteotomy and modular S-ROM prosthesis, resulting in acetabular cup implantation at the true acetabulum level, with excellent intramedullary filling of the prosthesis, and fixation effect of intramedullary nail at the proximal and distal osteotomies by the distal and proximal segments of the prosthesis. **c1**-**c2** At 3 months postoperatively, the AP pelvic and lateral femur radiographs showed that the femoral osteotomy was in the stage of osteophyte formation and the prosthesis stem was well filled. **d1**–**d2** At 9 months postoperatively, the AP pelvic and lateral femur radiographs showed that the acetabular and femoral prosthesis were stable, and the femoral osteotomy continued to heal. **e1**–**e2** AP hip radiographs at 3 years postoperatively showed good position of the prosthesis (without prosthesis subsidence), excellent healing of the osteotomy surfaces, and no limb-length discrepancy. **d** Lateral radiograph taken at 1 year postoperatively. Note: DDH, developmental dysplasia of the hip; PFD, proximal femoral deformity; AP, anteroposterior

The mean operation time was 102.6 ± 9.5 (range 90–112) min, and the mean intraoperative bleeding amount was 420 ± 31 (range 350–580) mL. No neurovascular injury or femoral fracture occurred intraoperatively. All 15 patients were followed up, with a mean follow-up duration of 62.5 (range 20–85) months. The postoperative limb-length discrepancy (1.0 ± 0.5 cm) was significantly less than the preoperative discrepancy (3.2 ± 1.2 cm), (*t* = − 2.501, *P* = 0.002). The Harris hip score was significantly improved from a mean of 47.2 ± 9.9 points preoperatively to 89.7 ± 3.9 points at the last follow-up visit (*t* = 21.31, *P* = 0.001). Immediate postoperative radiographs showed restoration of limb alignment after femoral osteotomy, excellent initial press-fit fixation of the S-ROM prosthesis, and good canal filling. According to Engh’s criteria [11], all 15 hips were graded as ingrown bones. Radiographs taken at 6 months postoperatively showed prosthesis subsidence in 2 hips (1.1 and 1.4 mm, respectively), which completely ceased from 1 year postoperatively. The mean duration of fracture healing was 6.2 (range 3.5–10) months (Fig. 2).

## Discussion

### Impact of PFD on hip arthroplasty

PFD can have a variety of causes and may have varying manifestations [1–5]. Accordingly, there are also a number of classification systems for PFD, including those proposed by the American Academy of Orthopedic Surgeons [12], D’Antonio et al. [13], and Berry [5]. Each classification system has its own specific focus and description with respect to the morphology and structure of PFD. However, no unified classification criteria are available and none of the existing systems provide a definitive clinical guidance for surgical techniques or prosthesis selection. Berry’s classification encompasses aspects such as the cause, site, and geometry of the deformity and is relatively more objective, detailed, and accurate than the other classification systems [5].

THA is a challenging procedure in the treatment of PFD owing to the abnormal anatomy of bones and soft tissues surrounding the hip joint. Thus, special THA techniques are required for patients with PFD [1, 3, 4]. The difficulties of this surgical technique include the following: (1) safe and accurate prosthesis implantation remains difficult when the morphology and anatomy of the proximal femur are abnormal; (2) it is difficult to obtain a femoral offset and limb length that is equal to the contralateral limb through surgical reduction; (3) it is challenging to achieve optimal hip joint reduction after intraoperative prosthesis implantation because of PFD-complicated soft-tissue spasm surrounding the hip joint; and (4) imbalanced muscle strength around the hip joint causes excessive wearing of the articular surface and poor stability of the prosthesis, resulting in postoperative hip dislocation. At present, femoral prostheses for initial replacement are mostly designed with a tapered stem, which requires a straight and patented proximal femoral canal to complete the implantation, as well as a fit between the stem shape and proximal femoral canal morphology [14]. For bioprostheses, immediate initial stability requires a press-fit between the stem and the canal [15, 16]. However, the proximal femoral anatomy and the canal morphology are both changed dramatically by PFD, especially the subtrochanteric rotational deformity, making it difficult to successfully implant the stem and greatly increasing the surgical difficulty of THA [1, 3, 4].

### PFD osteotomy and choices for prosthesis fixation

Osteotomy at the deformity site is often required for the successful implantation of the prosthesis stem during THA for PFD, especially at the subtrochanteric deformity [1, 2]. The surgical targets are as follows: (1) correction of malalignment through osteotomy at most sites with severe PFDs, (2) restoration of canal continuity for a successful prosthesis stem implantation, (3) good coaptation of the osteotomy surfaces, and (4) spanning of femoral prosthesis over the osteotomy ends and stable intramedullary fixation, both proximally and distally. There are many subtrochanteric osteotomy methods, such as the Becker V-shaped osteotomy, Paavilainen Z-shaped osteotomy [17], Anwar oblique osteotomy [18], and Reikeraas transverse osteotomy [2]; however, transverse osteotomy is the most practical and effective method for treating subtrochanteric rotational deformities. Usually, monosegmental osteotomy is the most preferred technique for sites that have been severely deformed by subtrochanteric deformities, whereas multisegmental osteotomy is a more surgically complex approach that increases the difficulty of prosthesis stem implantation and negatively affects bone healing [1–3, 6, 7].

In the past, cement spilling at the osteotomy surfaces might have occurred after cement-prosthesis fixation, affecting the bone-healing process [2]. Currently, bioprosthesis implantation is the preferred choice to ensure excellent femoral fixations both distally and proximally [1, 6, 8], which also requires an accurate fit between the prosthesis stem and the morphology of the distal and proximal femoral canals for the initial stability of the prosthesis. If there is good consistency and compliance in the morphology of the distal and proximal femoral canals after the coaptation of the osteotomy surfaces, then the selection of tapered bioprosthesis as an initial implant can achieve good fixation of the distal and proximal femur, whereas antegrade prosthesis insertion remains simple and feasible. However, a minimum overlapping of 7 cm of the prosthesis at the osteotomy end distally is required for adequate distal fixation length. When the consistency and compliance of the morphology of the distal and proximal femoral canals are suboptimal after osteotomy, then a complete fit between the prosthesis stem and the distal and proximal femoral canals remains difficult to achieve if a common tapered bioprosthesis is used. To address the morphological inconsistencies between the distal and proximal femoral canals and the poor fit between the stem and osteotomy surfaces, Togrul et al. [19] longitudinally split the proximal femur or placed one cortical bone around the pegged prosthesis in the canal of the distal fragment to achieve distal fixation. Paavilainen et al. [17] suggested the use of a distal fixation bioprosthesis stem after femoral osteotomy to obtain rotational stability of the distal femur via distal spanning of a thicker prosthesis stem over the osteotomy site. However, as most distal fixation prostheses have an in-built anteversion to accommodate the anterior bowing of the femur, antegrade prosthesis insertion might be susceptible to the risks of malcoaptation of the osteotomy surfaces and intraoperative femur fractures. Meanwhile, PFD is often complicated with abnormal femur anteversion, making it difficult to achieve large intraoperative adjustments of femur anteversion by using a common bioprosthesis for restoring normal alignment between the femoral head and the acetabulum.

For subtrochanteric PFD, osteotomy at the most severely deformed sites of the femur has been performed and a modular S-ROM prosthesis has been used for femoral reconstruction and fixation during THA. The modular S-ROM prosthesis consists of a porous-coated proximal component and a cylindrical polished femoral stem. The press-fit fixation between the proximal component and the elliptical section of the proximal femur enables more effective proximal femoral fixation. The distal cylindrical stem is inserted from the central hole of the proximal component by crossing the osteotomy end and is then fixed in the distal femoral canal. As the femoral canal that passes through the subtrochanteric transition zone to the canal isthmus is generally cylindrical and the cortical bone is thick [20], insertion of a cylindrical prosthesis into the distal femoral canal resembles the effect of a proximal femoral intramedullary nail. This, in turn, connects the distal and proximal femoral segments similarly to intramedullary fixation. Although a few patients may experience nonunion at the osteotomy site [10] or fracture of the modular prosthesis [21], bone grafting with longitudinally split cortical grids from the transected bones that envelop the osteotomy surfaces can effectively reduce the incidence of nonunions at the osteotomy site [22].

### Intraoperative and postoperative attention and experience

Whether the femur needs osteotomy and how to perform osteotomy have been controversial. It is believed that nonintercalation can achieve the purpose of reduction through preoperative high-weight traction and extensive soft-tissue release during surgery; however, it is difficult to reconstruct the acetabulum at the true level without osteotomy. It is generally believed that when the limb is extended to > 4 cm, the risk of blood vessel and nerve damage is increased. In patients with proximal femoral malformations, accurate classification is needed to fully understand the etiology of proximal femoral malformations; to assess the location, shape, and extent of the deformities; to select the combination of prosthesis in a targeted manner; and to formulate the main scheme and backup side for the malformations.

In the operation, osteotomy and orthopedic procedures are required. To avoid the cleft palate, the wire should be pre-tied at the osteotomy end. After the successful implantation of the S-ROM prosthesis, the pre-tied steel wire can be removed according to the stability of the fracture end. In these patients, the overall incidence of nerve injury was reported to be 1–2%. The highest incidence of clinically symptomatic sciatic nerve injury was 5.2%, which was 4 times that of other primary replacement surgery (1.3%). Moreover, 41.0% of the patients completely recovered after surgery, 44% had mild neurological dysfunction, and 15.2% had a poor prognosis, residual limb movement disorder, and persistent lower-limb numbness. Therefore, the tension of the sciatic nerve must be checked when the trial is reset. If the difficulty of reduction or the tension of the sciatic nerve is significantly increased, the osteotomy should be shortened. It is necessary for the surgeon to be familiar with the patient’s anatomy and the normal anatomical landmarks and to correctly position the anteversion angle of the prosthesis.

Some researchers have suggested that patients with PFD can undergo hip traction reduction before undergoing THA to reduce the height of femoral head dislocation before surgery, relax the contracted soft tissue of the hip, increase neurovascular tolerance, and reduce postoperative complications. However, in the treatment, we found that by retaining the gluteus medius and loosening the soft tissue of the hip (including the external rotation of the hip), the anterior and posterior joint capsules of the hip are loosened in the same incision and the iliopsoas muscle is prolonged. With the above method, the femoral head has not yet descended to the true sputum, and the combined femoral osteotomy can be reduced to the true level. None of the patients in this study underwent traction before surgery. The first phase of surgery was completed, reducing trauma and hospital stay.

## Conclusion

In conclusion, for patients with PFD, especially rotational PFD, careful assessments of the PFD location and morphology, as well as precise planning of the site and length of osteotomy, are required in THA. Osteotomy should be performed at the most severely deformed sites. The choice of a modular S-ROM prosthesis that is capable of segmental reaming and with a distal cylindrical design can assist in achieving stable proximal and distal femoral fixations during femoral reconstruction and fixation. Precise osteotomy and correct choice of the prosthesis are the key factors for achieving excellent postoperative outcomes. Our technique still requires investigation with respect to long-term efficacy in future studies.

## Data Availability

The data and materials are available from the medical records department of the 940th Hospital of PLA Joint Logistics Support Force. The datasets used and analyzed during the current study are available from the corresponding author on reasonable request.

## Abbreviations

AP: Anteroposterior
DDH: Developmental dysplasia of the hip
PFD: Proximal femoral deformity
THA: Total hip arthroplasty
